# The First Comprehensive Phylogeny of *Coptis* (Ranunculaceae) and Its Implications for Character Evolution and Classification

**DOI:** 10.1371/journal.pone.0153127

**Published:** 2016-04-04

**Authors:** Kun-Li Xiang, Sheng-Dan Wu, Sheng-Xian Yu, Yang Liu, Florian Jabbour, Andrey S. Erst, Liang Zhao, Wei Wang, Zhi-Duan Chen

**Affiliations:** 1 State Key Laboratory of Systematic and Evolutionary Botany, Institute of Botany, Chinese Academy of Sciences, Beijing, 100093, China; 2 University of Chinese Academy of Sciences, Beijing, 100049, China; 3 College of Life Sciences, Shanxi Normal University, Linfen, 041004, China; 4 Department of Ecology and Evolutionary Biology, University of Connecticut, Storrs, Connecticut, 06269–3043, United States of America; 5 Muséum national d’Histoire naturelle, Institut de Systématique, Evolution, Biodiversité, UMR 7205 ISYEB MNHN/CNRS/UPMC/EPHE, Sorbonne Universités, Paris, 75005, France; 6 Central Siberian Botanical Garden of the Siberian Branch of Russian Academy of Sciences, Zolotodolinskaya str. 101, Novosibirsk, 630090, Russia; 7 Laboratory of Systematics and Phylogeny of Plants, National Research Tomsk State University, prospekt Lenina 36, Tomsk, 634050, Russia; 8 College of Life Sciences, Northwest A&F University, Yangling, Shaanxi, 712100, China; Muséum national d'Histoire naturelle, FRANCE

## Abstract

*Coptis* (Ranunculaceae) contains 15 species and is one of the pharmaceutically most important plant genera in eastern Asia. Understanding of the evolution of morphological characters and phylogenetic relationships within the genus is very limited. Here, we present the first comprehensive phylogenetic analysis of the genus based on two plastid and one nuclear markers. The phylogeny was reconstructed using Bayesian inference, as well as maximum parsimony and maximum likelihood methods. The Swofford-Olsen-Waddell-Hillis and Bayesian tests were used to assess the strength of the conflicts between traditional taxonomic units and those suggested by the phylogenetic inferences. Evolution of morphological characters was inferred using Bayesian method to identify synapomorphies for the infrageneric lineages. Our data recognize two strongly supported clades within *Coptis*. The first clade contains subgenus *Coptis* and section *Japonocoptis* of subgenus *Metacoptis*, supported by morphological characters, such as traits of the central leaflet base, petal color, and petal shape. The second clade consists of section *Japonocoptis* of subgenus *Metacoptis*. *Coptis morii* is not united with *C*. *quinquefolia*, in contrast with the view that *C*. *morii* is a synonym of *C*. *quinquefolia*. Two varieties of *C*. *chinensis* do not cluster together. *Coptis groenlandica* and *C*. *lutescens* are reduced to *C*. *trifolia* and *C*. *japonica*, respectively. Central leaflet base, sepal shape, and petal blade carry a strong phylogenetic signal in *Coptis*, while leaf type, sepal and petal color, and petal shape exhibit relatively higher levels of evolutionary flexibility.

## Introduction

**Fig 1:**
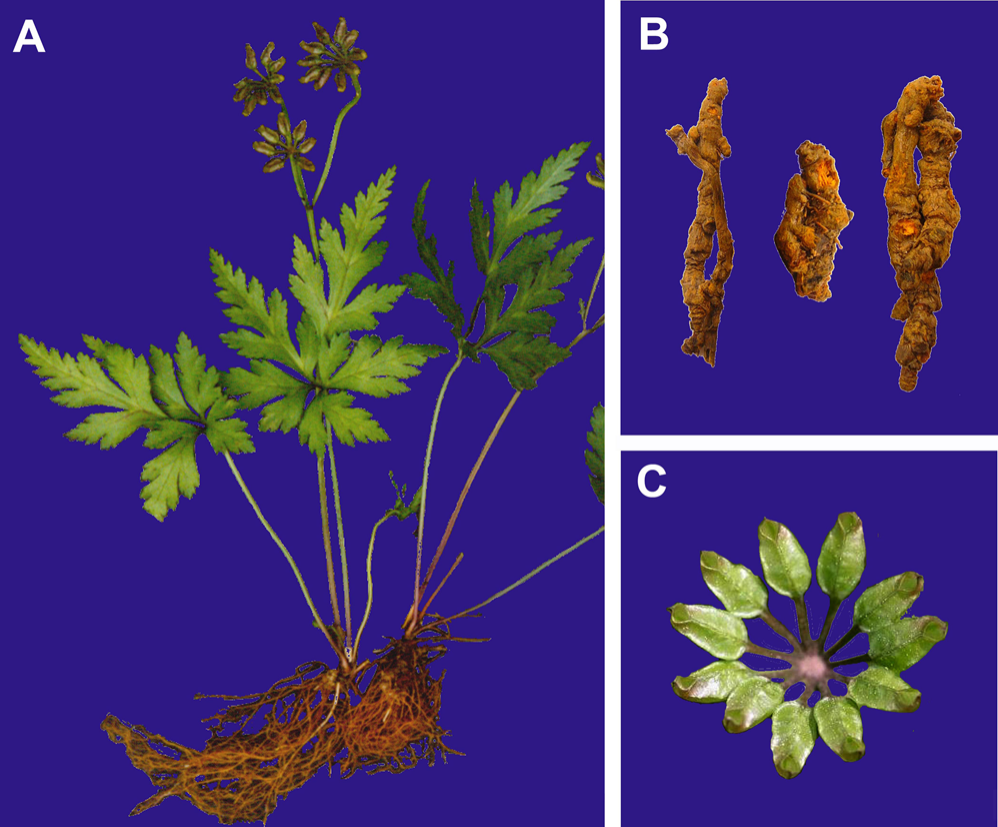
*Coptis chinensis*. A, plants; B, dried rhizome (*Rhizoma Coptidis*); C, carpels.

*Coptis* (Ranunculaceae) is one of the pharmaceutically most important plant genera in eastern Asia. Dried rhizomes of *Coptis* plants are utilized for *Rhizoma Coptidis* (RC), a traditional Chinese medicine famous for its functions of clearing heat, dispelling dampness, and purging fire toxins [1]. *Coptis* plants were for the first time recorded in the earliest monograph on Chinese material medica, *Sheng Nong’s Herbal Classic*, in the eastern Han dynasty (25–220 AD), and they have been used in many Chinese herbal medicines for more than two thousand years. *Coptis chinensis* (Fig 1A) has been widely cultivated in China and its rhizomes (Fig 1B) are largely exported to other countries. In Korea and Japan, the rhizomes of *C*. *japonica* are sometimes used as a substitute to that of *C*. *chinensis* [2]. Since the 18^th^ century, Native Americans have used the rhizome of *C*. *trifolia* to treat mouth sores, poor digestion and infections [3]. Phytochemical and pharmacological studies on *Coptis* plants indicate that they contain a number of alkaloids, such as berberine, palmatine, jatrorrhizine, coptisine, columbamine, and epiberberine [4,5]. RC has been shown to have various clinical effects, such as suppression of fever, cessation of dampness, detoxification [6], and antibacterial, antiviral, antiinflammatory, and antihyperglycemic activities [7–9]. Recent studies have also indicated that berberine and jatrorrhizine extracted from RC have potential therapeutic implications for the treatments of obesity [10] and hypercholesterolemia [11], respectively. In marked contrast to the extensive knowledge about the pharmacological properties of *Coptis*, understanding of the evolution of morphological characters and phylogenetic relationships within the genus is extremely limited.

*Coptis* is characterized by a whorl of stipitate carpels that are not totally closed at the apex ([12]; Fig 1C) and double median bundles in the petioles, petiolules and laminae midribs [13]. In addition, *Coptis* plants lack cauline leaves (Fig 1A). Although the monophyly of *Coptis* is not disputed, the circumscription of some species remains problematic, namely *C*. *morii*, *C*. *groenlandica*, and *C*. *lutescens*. The individuals of *Coptis* endemic to Taiwan were first described as *C*. *morii* by Hayata [14]. Yoshimatsu and Yamamoto subsequently considered *C*. *morii* to be synonymous to Japanese *C*. *quinquefolia* [15]. Chinese authors accepted this synonymy [16–18], but Japanese authors still supported the separate species status of *C*. *morii* [19,20]. *Coptis groenlandica* was published by Fernald for individuals from Greenland and eastern North America [21]. Hultén treated the species as a subspecies of *C*. *trifolia* from Alaska and eastern Asia, *C*. *trifolia* subsp. *groenlandica* [22]. Later, Hultén farther synonymised it under *C*. *trifolia* [23], which was accepted by most authors [19,24]. The description of *C*. *lutescens* highlighted many morphological similarities with *C*. *japonica* [25]. The latter contains three to four varieties [26,27]. In the most recent worldwide monograph of Ranunculaceae [19], the genus consists of 15 species, of which six occur in China, six in Japan and Far Eastern Siberia, and four in North America (Table 1). Due to overharvesting and/or loss of habitat caused by human activities, many species in *Coptis* have become endangered or their population sizes have declined. For example, wild *Coptis* plants are scarce in Mainland China, and all five species and one variety in Mainland China are listed in the national key preserved wild plants [28].

**Table 1:** Summary of the taxonomic history of *Coptis*, showing the main systems of classification.

Torrey and Gray [29]^1^	Satake [30]^2^	Tamura [19]
Sect. *Chryza*	Subgen. *Chryza*	Subgen. *Coptis*
*C*. *trifolia*	*C*. *trifolia*	*C*. *trifolia*
	Subgen. *Metacoptis*	Subgen. *Metacoptis*
–	Sect. *Japonocoptis*	Sect. *Japonocoptis*
	*C*. *quinquefolia*, *C*. *trifoliolata*	*C*. *morii*, *C*. *quinquefolia*, *C*. *ramosa*, *C*. *trifoliolata*
Sect. *Chrysocoptis*	Sect. *Chrysocoptis*	Sect. *Chrysocoptis*
*C*. *occidentalis*	*C*. *japonica*	2 species in Japan (*C*. *japonica*, *C*. *lutescens*), 5 in Mainland China (*C*. *chinensis*, *C*. *deltoidea*, *C*. *omeiensis*, *C*. *quinquesecta*, *C*. *teeta*), and 3 in North America (*C*. *aspleniifolia*, *C*. *laciniata*, *C*. *occidentalis*)
Sect. *Pterophyllum*	–	
*C*. *asplenifolia*		

1 = Torrey and Gray included only taxa from North America [29].

2 = Satake included only taxa from Japan [30].

**Fig 2:**
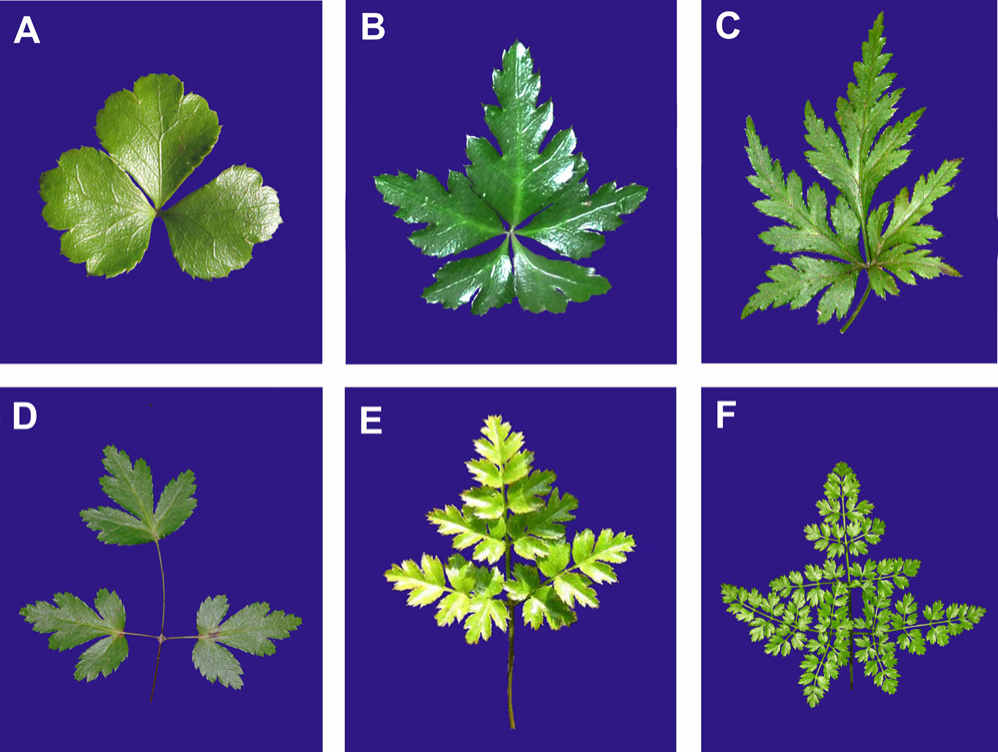
Leaf diversity of *Coptis*. A. *C*. *trifolia*; B. *C*. *morii*; C. *C*. *chinensis*; D. *C*. *laciniata*; E. *C*. *aspleniifolia*; F. *C*. *japonica*.

**Fig 3:**
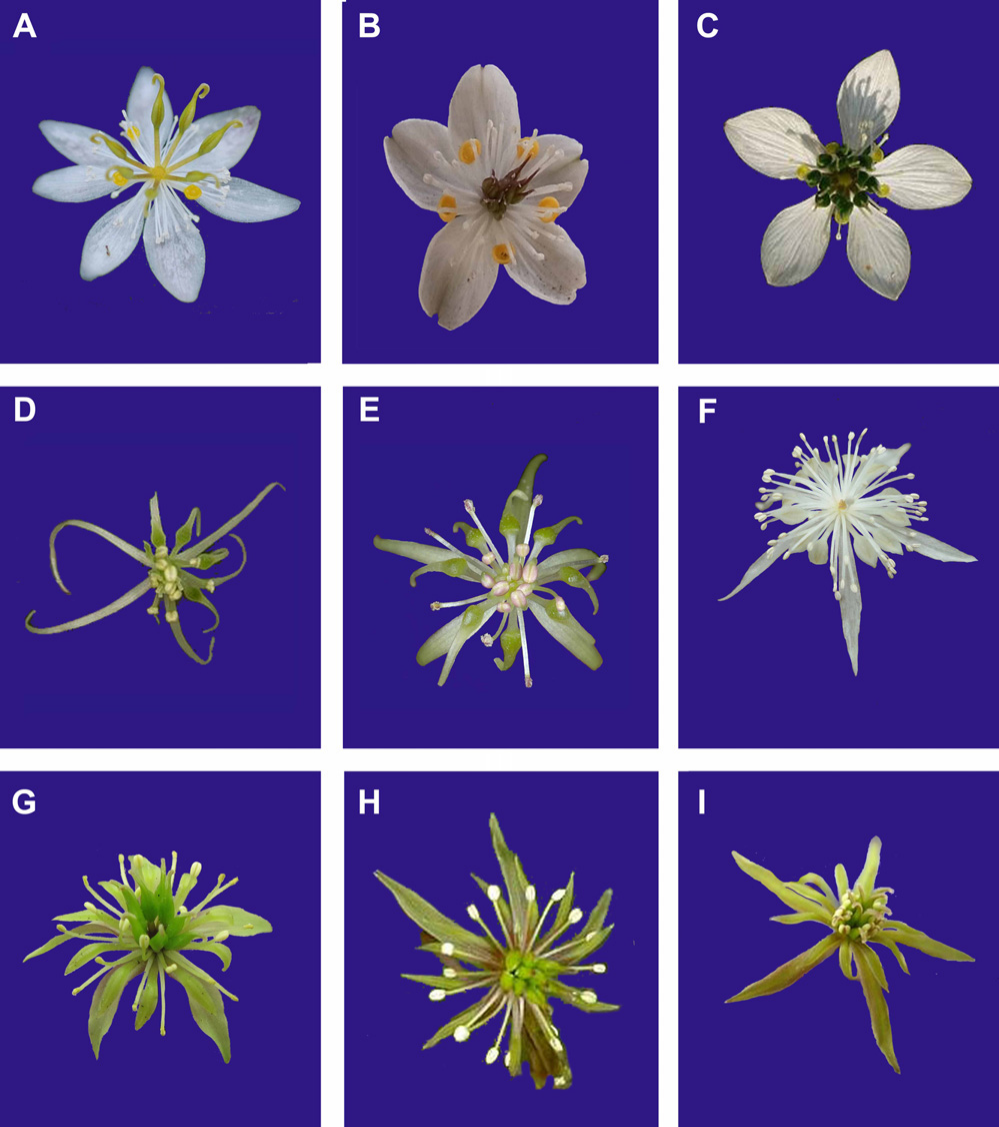
Floral diversity of *Coptis*. A. *C*. *trifolia*; B. *C*. *quinquefolia*; C. *C*. *morii*; D. *C*. *asplenifolia*; E. *C*. *laciniata*; F. *C*. *japonica*; G. *C*. *chinensis*; H. *C*. *deltoidea*; I. *C*. *omeiensis*.

The taxonomy of *Coptis* has traditionally been based on vegetative (leaf type and shape; Fig 2) and reproductive (flower number, color and shape of sepals and petals, and beak length; Fig 3) characters. Several local or worldwide infrageneric classifications have been proposed for *Coptis* based on morphological characters (Table 1). Torrey and Gray classified American species of *Coptis* into three sections, namely *Chryza*, *Chrysocoptis* and *Pterophyllum* [29]. Satake revised the Japanese species and classified them into two subgenera, *Chryza* and *Metacoptis* (including two sections, *Chrysocoptis* and *Japonocoptis*) [30]. Integrating the above two classifications, Tamura put forward the first worldwide classification of this genus, in which *Coptis* was subdivided into two subgenera, *Coptis* (= *Chryza* sensu Torrey and Gray) and *Metacoptis*, with the latter further subdivided into three sections [31]. Tamura merged section *Pterophyllum* into section *Chrysocoptis* [19]. However, the infrageneric classification of *Coptis* has not been evaluated in a phylogenetic context so far.

Phylogenetic studies of Ranunculaceae [32–36] resulted in the recognition of the monophyletic Coptidoideae comprising *Coptis* and the monotypic genus *Xanthorhiza*. Recently, a molecular study of *Coptis* has been published, in which 13 *Coptis* species and two outgroup taxa from Ranunculoideae were included, and only four species, sampled from Chinese Mainland, were sequenced for six plastid regions, whereas all others were sequenced for the plastid *rbcL* gene only [37]. This study led to the conclusion that the four species of Mainland China formed a clade, but provided no insights into the phylogeny of the genus as a whole.

In our study, two plastid (*trnL-F* and *trnH-psbA* spacers) and one nuclear (ITS) DNA regions were used. Our aims are (1) to provide the first comprehensive molecular phylogenetic framework for *Coptis* containing all 15 species recognized by Tamura [19]; (2) to test the monophyly of infrageneric groupings recognized in classifications of *Coptis*; (3) to clarify the systematic status of *C*. *morii*, *C*. *groenlandica*, and *C*. *lutescens*; and (4) to interpret evolution of key morphological characters in a phylogenetic context.

## Materials

### Ethics statement

This study did not require any special permits because all collecting was performed by researchers located at institutes with the permits required such as IBCAS (Institute of Botany, Chinese Academy of Sciences) in Beijing. One plant sample was collected in Mt. Poluo, Yilan county, Taiwan, in the company of the staff of Taiwan Forestry Research Institute (SWC). Eight are herborized materials deposited in the Herbarium, Institute of Botany, the Chinese Academy of Sciences, Beijing (PE).

### Taxon sampling and DNA sequencing

A total of 21 individuals were sampled, representing all 15 *Coptis* species recognized by Tamura [19] and *C*. *groenlandica*. We selected *Xanthorhiza simplicissima* as an outgroup because it was recognized as sister to *Coptis* by previous studies [33,36,38]. The outgroups also included *Megaleranthis saniculifolia* from the Ranunculoideae, related to the Coptidoideae [36]. Vouchers and GenBank accession numbers are listed in S1 Table.

Total genomic DNA was extracted from fresh silica gel-dried leaves or herbarium specimens using DNeasy Mini Plant Kits (Tiangen Biotech, Beijing, China) following the manufacturers’ protocol. The selected DNA regions were amplified with standard polymerase chain reaction (PCR). The *trnL-F* spacer was amplified using the *e* and *f* primers of Taberlet et al. [39] and PCR conditions described by Li et al. [40]. The *trnH-psbA* spacer was amplified using the primers and PCR conditions recommended by Shaw et al. [41]. For the ITS region, we used the same primers and conditions as Chen and Li [42]. All PCR products were purified using the Tian quick Midi Purification Kit (Tiangen Biotech) following the manufacturer’s protocol. Sanger sequencing was conducted on an ABI 3730xl DNA sequencer. Geneious v6.0 [43] was used to edit chromatograms and contigs.

### Phylogenetic analysis

The resulting sequences were initially subjected to a BLAST search against GenBank database (www.ncbi.nlm.nih.gov) to test for potential contamination and to confirm the targeted markers. Sequences were aligned using Clustal X v1.83 [44] and manually adjusted with BioEdit v7.0 [45]. Three poly T regions in the *trnL-F* matrix (encompassing 28 positions) and one poly T region in the *trnH-psbA* matrix (12 positions) were excluded from the analyses.

Phylogenetic analyses of the plastid (*trnL-F* and *trnH-psbA*), ITS, and combined plastid and ITS datasets were carried out using Bayesian inference (BI) method in MrBayes v3.2.5 [46]. For all Bayesian analyses, each DNA region was assigned its own model of nucleotide substitution, as determined by the Akaike Information Criterion (AIC) via jModeltest v2.1.4 [47]. Four Markov Chain Monte Carlo chains (three incrementally heated and one cold) were independently run twice, sampling one tree every 1000 generations for 50 million generations, starting with a random tree. Completion was determined by the average standard deviation of split frequencies falling below 0.01. The stationarity of the runs was assessed using Tracer v1.5 [48]. Majority rule (>50%) consensus trees were constructed after removing the burn-in period samples (the first 25% of sampled trees). The posterior probability (PP) values were calculated using the default procedure and were used as an estimate of nodal robustness. For the purpose of comparison and confirmation, we also performed phylogenetic analyses using maximum parsimony (MP) and maximum likelihood (ML) methods in PAUP* v4.0b10 [49] and RAxML v7.0.4 [50], respectively. Nodes with PP ≥ 0.95 [51] and BS ≥ 70% [52] were considered to be well supported.

Additionally, we calculated sequence divergence in each molecular dataset by Kimura’s 2-parameter method [53] in MEGA v6.06 [54]. Outgroups were deleted in this analysis.

### Alternative hypothesis test

We used two methods, the Swofford-Olsen-Waddell-Hillis (SOWH) test [55] and the Bayesian test [56], to assess the strength of the conflicts between the traditional taxonomic units and those recognized by the phylogenetic inferences. The alternative hypotheses are: 1) subgenus *Metacoptis* is monophyletic, 2) *C*. *japonica* is monophyletic, and 3) *C*. *chinensis* is monophyletic. For the SOWH test, we first constrained the tree such that a specific group as monophyletic, and optimized the tree topology, branch lengths and tree scores using RAxML v7.0.4 [50] from the original data (GTR + Γ model and partitioned by DNA region). We then simulated 100 replicate datasets using Seq-Gen v1.3.2 [57]. For each simulated dataset, ML searches were conducted under “optimal constrained” and “optimal unconstrained” conditions. The distribution of log likelihood differences (ΔlnL) was used to evaluate the significance of the difference between the unconstrained tree and the constrained hypothetical tree [58].

The Bayesian test was performed by following the approach of Kass and Raftery [56]. We first run Bayesian analyses in MrBayes v3.2.5 [46], with each hypothetical topology constrained. Convergence to stable values was checked in Tracer v1.5 [48] and the effective sample size (ESS) value of each parameter was greater than 200 (after excluding a burn-in fraction of 25%). The harmonic mean marginal likelihood was obtained from each constrained analysis, and compared to the one obtained from the optimal analysis. The Bayes Factor (BF) is calculated as the twice difference between optimal and constrained harmonic means. We used the thresholds of Kass and Raftery [56], 2ln BF > 2, as an indication of significant difference between the constrained and unconstrained trees.

### Morphological characters

The distribution of 19 discrete vegetative and reproductive characters, on which the taxonomy of *Coptis* has been mostly based, is indicated in S2 Table. Information on morphological features was extracted from basic floras [16,24,27] and was completed by personal observations. For the 2- to 4-ternate and pinnate compound leaves, lateral leaflet base was only scored for the first leaflet. Information on some characters is missing for some species and we consequently inferred the evolution of two vegetative (leaf type and type of central leaflet base) and six floral (color and shape of sepals and petals, petal number, and type of petal blade) characters with the aim of illustrating these morphological context underlying the phylogenetic hypothesis presented here. In this analysis, we used *Xanthorhiza* as an outgroup, owing to its sister relationship to *Coptis*. The inference of character evolution was performed using a series of Bayesian reversible-jump hyperprior (RJHP) MCMC analyses [59] in BayesTraits v2.0 [60] on 1000 randomly chosen posterior trees. All Bayesian analyses used 25 million generations, with sampling every 1000 generations. The stationarity of the runs was assessed using Tracer v1.5 [48] and the first 25% generations were discarded.

We also tested for correlated evolution between each pair of characters using BayesTraits v2.0 [60] using RJHP MCMC with the same methods just described. The ESS values for all other parameters are greater than 200 except for the ESS of the harmonic mean (< 100). We implemented the independent contrast module, which investigates correlated evolution between a pair of traits by comparing the harmonic mean marginal likelihood of two models for independent *vs*. dependent evolution of traits. The BF is calculated as the twice difference between dependent and independent harmonic means. When 2ln BF < 2, the pair of characters is weakly correlated; 2ln BF > 2 represents positive correlation [56].

## Results

### Characteristics of *trnL-F*, *trnH-psbA*, and ITS sequences

The aligned *trnL-F*, *trnH-psbA*, and ITS matrices included 449, 360, and 700 characters, respectively. The characteristics of the three molecular datasets are summarized in Table 2. Within *Coptis*, *trnL-F* sequence divergence ranges from 0.0% to 2.5% (S3 Table); *trnH-psbA* sequence divergence ranges from 0.0% to 4.5% (S4 Table); and ITS sequence divergence ranges from 0.0% to 13.5% (S5 Table).

**Table 2:** Statistics from the analyses of the various datasets.

Data set	No. taxa	Total length	No. variable characters	No. informative characters	No. trees	Length of trees	CI	RI	RC	Model
*trnL-F*	21	449	53	15	10	58	0.95	0.93	0.88	GTR + Γ
*trnH-psbA*	22	360	93	19	600	106	0.96	0.94	0.91	GTR + Γ
plastid DNA	23	809	146	34	79	166	0.95	0.92	0.87	
ITS	18	700	140	92	6	233	0.73	0.76	0.58	GTR + Γ
Combined	23	1509	286	126	18	403	0.81	0.82	0.67	

Abbreviations: CI, consistency index; RI, retention index; RC, rescaled consistency index.

### Phylogenetic analyses

**Fig 4:**
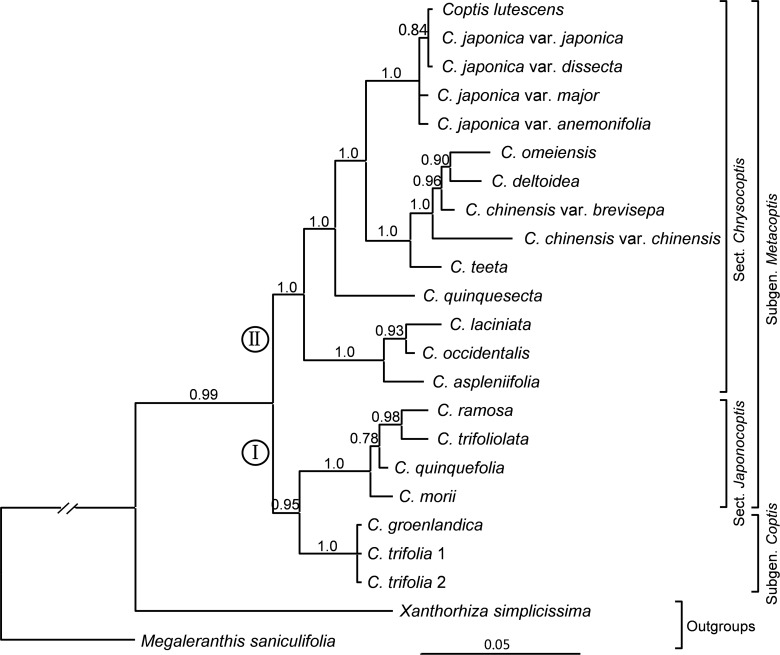
Bayesian phylogram inferred from the combined plastid DNA and ITS data. Numbers above the branches are Bayesian posterior probabilities. Tamura’s [19] classification is shown on the right.

No conflicting nodes with strong support (≥ 70% BS and ≥ 0.95 PP) were found between the plastid and ITS trees (S1 and S2 Figs). The combined plastid and ITS dataset consisted of 1509 characters. BI and ML analyses resulted in identical trees that were congruent with the MP trees except that one node was collapsed in the consensus tree (Fig 4; S3 Fig). The tree based on the combined plastid and ITS dataset is highly congruent with the plastid and ITS trees, and shows higher internal nodal supports. All further discussion will be based on the combined plastid and ITS phylogenetic hypothesis (Fig 4).

*Coptis* is strongly supported as monophyletic (PP = 0.99). Within *Coptis*, two major clades (I and II) are identified. Clade I consists of subgenus *Coptis* and section *Japonocoptis* of subgenus *Metacoptis* (PP = 0.95). Within section *Japonocoptis*, *C*. *morii* is sister to the remainder (PP = 0.78). *Coptis groenlandica* and the two *C*. *trifolia* accessions formed a polytomy. Clade II consists of section *Chrysocoptis* of subgenus *Metacoptis*. Within clade II, three North American species form the earliest-diverging lineage (PP = 1.0), followed by *C*. *quinquesecta* (PP = 1.0). *Coptis lutescens* is nested in *C*. *japonica* and is grouped with *C*. *japonica* var. *japonica* and *C*. *japonica* var. *dissecta* (PP = 0.84). *Coptis chinensis* var. *chinensis* is sister to the subclade containing *C*. *chinensis* var. *brevisepala*, *C*. *omeiensis* and *C*. *deltoidea* (PP = 0.96).

### Alternative hypothesis test

**Fig 5:**
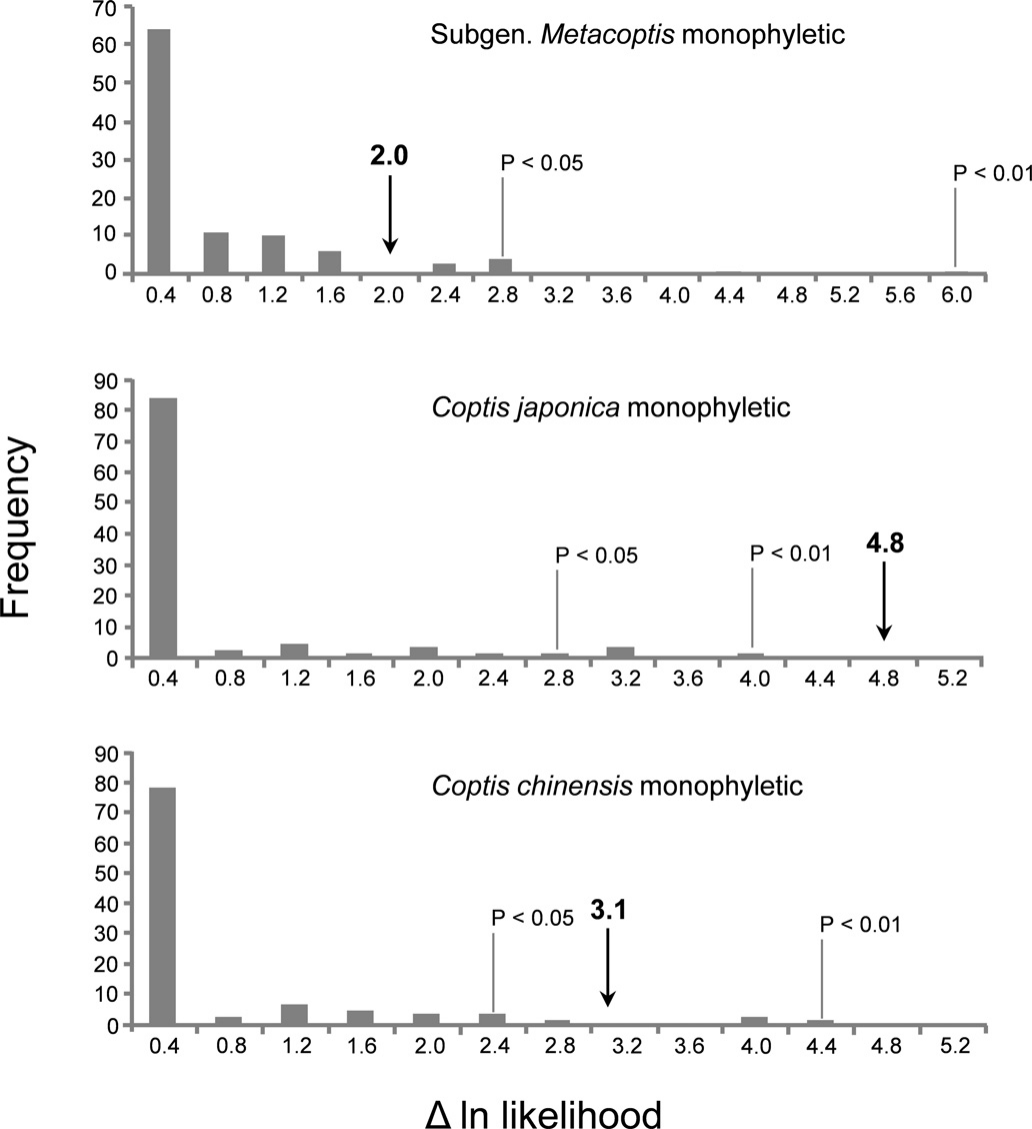
The distributions for the SOWH test of the three alternative topologies. The histogram shows the distribution of 100 replicates. The 1%, 5% significance levels and the observed log-likelihood difference are shown for each hypothesis in the chart (See text for details).

The results of the SOWH tests for three alternative hypotheses are shown in Fig 5. Constraining *C*. *japonica* as monophyletic yielded a ln-likelihood that is 4.8 units worse than the unconstrained optimal tree. This difference is significant at the 0.01 level (4.0 units), the monophyly of *C*. *japonica* should thus be rejected. The monophyly of *C*. *chinensis* is also rejected by the SOWH test (3.1 *vs*. 2.4 at the 0.05 level). The monophyly of subgenus *Metacoptis* could not be rejected by the SOWH test (2.0 < 2.8 at the 0.05 level). Similarly, the Bayesian tests rejected the monophyly of *C*. *japonica* and *C*. *chinensis*, but not of subgenus *Metacoptis* (Table 3).

**Table 3:** Bayesian tests on the alternative hypotheses.

Hypothesis	HM marginal likelihood	2ln BF
Subgen. *Metacoptis* monophyletic	-4069.49	–
*Coptis japonica* monophyletic	-4074.00	3.76
*Coptis chinensis* monophyletic	-4077.86	5.31

Harmonic mean (HM) marginal likelihood obtained from the optimal unconstrained analysis is -4070.73.

### Reconstruction of character evolution

**Fig 6:**
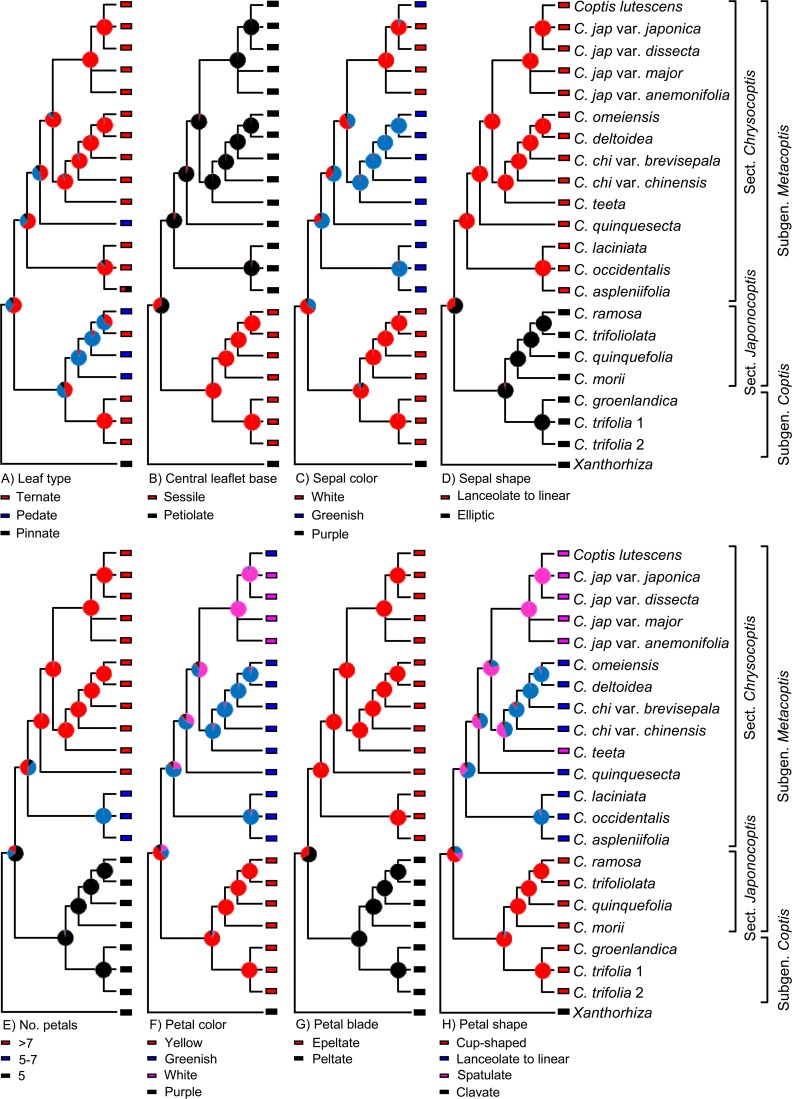
The evolutionary reconstruction of selected morphological characters in *Coptis*. Color-coded pie diagrams at each node show the relative probabilities of alternative ancestral states.

Evolutionary reconstructions of eight characters were indicated in Fig 6. Leaf type, sepal color, and petal color and shape are homoplastic in *Coptis*. For example, white sepals occur in two unrelated lineages, Clade I and *C*. *japonica* of Clade II. Petal number has an increment from 5 to approximately 10. Central leaflet base, sepal shape, and petal blade correlate well with the phylogeny generated from the combined plastid and ITS dataset.

The results of the tests of correlated evolution for these eight characters are listed in Table 4. The majority of the associations between pairs of the eight morphological characters are not statistically correlated. However, leaf type exhibits an unexpected pattern of correlated evolution with number of petals (2ln BF = 3.34). Petal color shows positively correlated with sepal color and petal shape and blade. In addition, central leaflet base shows positive patterns of correlated evolution with sepal shape.

**Table 4:** Probability of observed pattern of correlated evolution between pairs of characters.

	LT	CLB	SC	SS	NP	PC	PS	PB
Leaf type (LT)		–	-2.29	–	**3.34**	-0.58	–	-1.49
Central leaflet base (CLB)			–	**2.11**	1.78	-1.48	–	-0.78
Sepal color (SC)				–	–	**3.34**	-2.02	-1.45
Sepal shape (SS)					–	-0.39	1.03	0.46
No. petals (NP)						–	1.85	0.69
Petal color (PC)							**2.95**	**2.48**
Petal shape (PS)								–
Petal blade (PB)								

“–” indicates that the difference between dependent and independent harmonic means is negative. Bolds indicate a positive (2ln BF > 2) pattern.

## Discussion

### Major clades of *Coptis*

*Coptis* was traditionally first subdivided into two groups, namely *C*. subgen. *Coptis* and subgen. *Metacoptis*, based on the veins of the fruit lateral faces and beak length (Table 1). In subgenus *Coptis*, follicles have a longitudinal vein on each lateral face and a long beak, whereas in subgenus *Metacoptis*, as well as *Xanthorhiza*, follicles lack a longitudinal vein on each lateral face and have a relatively short beak [19,31]. Our phylogenetic analyses strongly suggest that subgenus *Coptis* is embedded in subgenus *Metacoptis* and is sister to section *Japonocoptis*, although the SOWH and Bayesian tests do not reject the monophyly of subgenus *Metacoptis*. Subgenus *Coptis* shares many morphological characters with section *Japonocoptis*, such as sessile central leaflets (Fig 2A and 2B), elliptic sepals, petals non-concolorous with sepals (Fig 3A–3C), and cup-shaped petals (Fig 6).

The monophyly of section *Chrysocoptis* (clade II) is strongly supported (Fig 4); it is characterized by petiolate central leaflets (Fig 2C–2F), lanceolate to linear sepals (Fig 3D–3I), and epeltate petals (Fig 6). Torrey and Gray erected the section *Pterophyllum* for *C*. *aspleniifolia* [29], whose petals are dilated and cucullate in the middle (Fig 3D). This section was first accepted by Tamura [31], and then rejected [19]. Our analyses strongly support that *C*. *aspleniifolia* is grouped with *C*. *occidentalis* and *C*. *laciniata* of section *Chrysocoptis* (Fig 4). These three species share some morphological characters, such as pale brown rhizomes, central and lateral leaflets with distinct petiolates, and petals filiformly attenuate upwards. Moreover, floral characters of *C*. *laciniata* (Fig 3E) are very similar with those of *C*. *aspleniifolia*.

### The taxonomic states of *C*. *morii*, *C*. *groenlandica*, *C*. *lutescens*, and *C*. *chinensis* var. *brevisepala*

Based on the collections of U. Mori at Taiwan, Mt. Rontabunzan (Hehuan) in April 1910, Hayata published *C*. *morii* and considered that this species was similar to the Japanese *C*. *quinquefolia*, but quite distinguishable from the latter by its much larger leaves [14]. Yoshimatsu and Yamamoto treated *C*. *morii* as a synonym of *C*. *quinquefolia* without any explanation [15], which was followed by Chinese authors [16–18]. Based on a comparative study, Tamura suggested that *C*. *morii* differs from *C*. *quinquefolia* in having branched scapes (*vs*. not) and stolons (*vs*. lacking), and differs from *C*. *ramosa* in leaf characters [61]. Currently, *C*. *morii* is considered a distinct species by Japanese authors [19,20]. Our analyses indicate that in section *Japonocoptis*, *C*. *morii* is early diverging, whereas *C*. *quinquefolia* is sister to *C*. *ramosa*-*C*. *trifoliolata*. The *trnH-psbA* sequence divergence is 1.5% between *C*. *morii* and *C*. *quinquefolia*, which is higher than that of 21 species pairs, such as *C*. *quinquefolia*-*C*. *ramosa* (0.7%), *C*. *quinquefolia-C*. *trifoliolata* (0.0%), and *C*. *quinquefolia*-*C*. *trifolia* (0.7%) (S4 Table). The ITS sequence divergence resulted in similar results (S5 Table). In this study, we also show that *C*. *morii* markedly differs from *C*. *quinquefolia* in flower number (4 *vs*. 1), bract shape (lanceolate *vs*. elliptic), and bract margin (acute serrate *vs*. entire). Therefore, our data support the species *C*. *morii* as segregate from the other species in section *Japonocoptis*.

In 1929, Fernald described individuals from Alaska and eastern Asia as *C*. *trifolia* and those from Greenland and eastern North America as *C*. *groenlandica*. Hultén treated them as subspecies, *C*. *trifolia* subsp. *trifolia* and *C*. *trifolia* subsp. *groenlandica* [22]. Seven years later, Hultén further synonymised *C*. *groenlandica* under *C*. *trifolia* [23]. However, Taylor and Mulligan considered that the two subspecies could be different in sepals and seed shape [62]. Based on a detailed comparative study of individuals from eastern Asia and North America, Ford suggested that no clear distinction could be made between the two taxa [24]. Comparing the *trnL-F* and ITS sequences, we did not find any divergence between *C*. *groenlandica* and the two accessions of *C*. *trifolia*. This result supports the hypothesis that both names are synonymous and designate the same species.

Tamura published *C*. *lutescens* and considered that it was distinguishable from *C*. *japonica* by yellowish flowers (*vs*. white) and narrower and acuminate sepals, and grows on mossy ground under subalpine or upper temperate conifer forests [25]. However, white flowers sometimes appear in populations of *C*. *lutescens* (S1 Table). The species in section *Chrysocoptis* have lanceolate to linear sepals and preferably grow on coniferous forest floors [16,27]. In our study, phylogenetic analyses indicate that *C*. *lutescens* is nested within *C*. *japonica* (Fig 4; S1–S3 Figs). The SOWH and Bayesian tests strongly reject the monophyly of *C*. *japonica* (Fig 5; Table 3). The *trnH-psbA* sequence divergence between *C*. *lutescens*-*C*. *japonica* var. *anemonifolia* (0.0%) is equal or inferior to the sequence divergence among the four varieties of *C*. *japonica* (S4 Table). Similarly, the ITS sequence divergence between *C*. *lutescens*-*C*. *japonica* var. *japonica* (0.0%) is lower than the sequence divergence among the four varieties of *C*. *japonica* (0.4%-1.6%) (S5 Table). Thus, our analyses support the inclusion of *C*. *lutescens* within *C*. *japonica*. To clarify that *C*. *lutescens* is a synonym or variety of *C*. *japonica*, further study at the population level is needed.

Our phylogenetic analyses do not group *C*. *chinensis* var. *chinensis* and *C*. *chinensis* var. *brevisepala* (Fig 4; S1–S3 Figs). The monophyletic *C*. *chinensis* is also rejected by the SOWH and Bayesian tests (Fig 5; Table 3). The ITS sequence divergence between *C*. *chinensis* var. *chinensis*-*C*. *chinensis* var. *brevisepala* (0.63%) is higher than the sequence divergence between *C*. *chinensis* var. *brevisepala*-*C*. *omeiensis* (0.28%), *C*. *chinensis* var. *brevisepala-C*. *deltoidea* (0.28%), and *C*. *omeiensi -C*. *deltoidea* (0.32%) (S5 Table). *Coptis chinensis* var. *brevisepala* differs from *C*. *chinensis* var. *chinensis* in sepal length and distribution [16,18]. The former has sepals slightly longer than petals (~ 6.5 mm), and is distributed in southern China, whereas the latter has sepals twice as long as petals (9–13 mm) and is found in central China. Therefore, our data suggest that *C*. *chinensis* var. *brevisepala* should be elevated to the species rank.

### Evolution of morphological characters

Leaf and flower characters have traditionally played a role in the taxonomy of *Coptis*. Here we discuss the implications of the molecular tree for their evolution (Fig 6; S2 Table).

Leaf morphology in *Coptis* is diverse, including leaf type and central leaflet base (Fig 2). Our data suggest that ternate compound leaf could be a synapomorphy of the genus (Fig 6A), and multi-ternate compound leaves appear in *C*. *japonica* (including *C*. *lutescens*), *C*. *laciniata* (sometimes 1-ternate), and *C*. *aspleniifolia* (mainly 2- to 3-pinnate). Pedate leaves occur in two different clades, section *Japonocoptis* and *C*. *quinquesecta* of section *Chrysocoptis*. Within a certain species, leaf type is sometimes variable, such as in *C*. *aspleniifolia* (S2 Table). In *C*. *morii*, leaves are usually described as pedate and have five leaflets (Fig 2E). Checking herbarium specimens in National Taiwan University, Taiwan, we also found one individual whose two lateral leaflets did not split entirely (Collect No. 2629), just like in *C*. *chinensis* (Fig 2F). Central leaflet base correlates well with our tree (Fig 6B). In clade I (including section *Japonocoptis* and subgenus *Coptis*), central leaflets are sessile, whereas in clade II (section *Chrysocoptis*) central leaflets have distinct petiolules. In *Coptis*, compound leaves occur, but leaflets lack petiolules or petiolules have no ventral bundle, which suggests that the compound leaves of *Coptis* are not well constituted [13].

The distribution of sepal and petal colors is partially coherent agreement with our phylogenetic hypothesis: clade I (including section *Japonocoptis* and subgenus *Coptis*) and *C*. *japonica* of clade II (section *Chrysocoptis*) have white sepals and yellow petals, whereas the remaining members of clade II have greenish white or greenish yellow sepals and petals (Fig 6C and 6F). Elliptic sepals and peltate petals are plesiomorphic in the genus, which occur in clade I and *Xanthorhiza*, whereas clade II has linear-lanceolate sepals or epeltate petals (Fig 6D and 6G). Species from clade I and *Xanthorhiza* have five petals, whereas in clade II, the earliest diverging lineage containing three North American species has 5–7 petals, and the remaining taxa have about 10 petals. Our data thus suggests an increment of petal number in a unidirectional manner (Fig 6E). Cup-shaped petals occur in clade I, whereas members of clade II have linear-lanceolate petals except *C*. *teeta* and *C*. *japonica* (including *C*. *lutescens*) which have spatulate petals (Fig 6H).

Our inferences of character evolution therefore indicate that morphological synapomorphies for subgenus *Coptis* and section *Japonocoptis* of subgenus *Metacoptis* (clade I) include epetiolate central leaflets and yellow and cup-shaped petals. The tests of correlated evolution shows the associations between pairs of these three characters are not positive (Table 4). The synapomorphies for section *Chrysocoptis* of subgenus *Metacoptis* (clade II) include lanceolate to linear sepals and epeltate petals. Sepal shape does not exhibit positively correlated with petal blade (Table 4). Based on these identified synapomorphies, the below adjustments for *Coptis* classification would thus be credible.

### *Coptis* classification

The most recent subgeneric and sectional classification of *Coptis* ([19]; Table 1) is partly supported by our plastid and ITS data, which recover the two sections of subgenus *Metacoptis* exactly as circumscribed by Tamura [19]. Subgenus *Coptis*, however, is embedded in subgenus *Metacoptis* and will need to be adjusted, while subgenus *Metacoptis* also needs adjustment to attain monophyly (namely the removal of section *Japonocoptis*). Additionally, *C*. *morii* is a species in its own right and *C*. *chinensis* var. *brevisepala* should be elevated to a species rank, whereas *C*. *groenlandica* and *C*. *lutescens* need to be abandoned.

## Supporting Information

S1 Fig ML inferred from the plastid DNA data. The results of MP and ML bootstrap analyses are shown above the branches, whereas the values below the branches result from Bayesian analysis. “*” indicates the nodes not found in the strict consensus tree. Tamura’s [19] classification is shown on the right.
(TIF)

S2 Fig ML inferred from the ITS data. The results of MP and ML bootstrap analyses are shown above the branches, whereas the values below the branches result from Bayesian analysis. “*” indicates the node not found in the strict consensus tree. Tamura’s [19] classification is shown on the right.
(TIF)

S3 Fig ML tree inferred from the combined plastid DNA and ITS data. The results of MP and ML bootstrap analyses are shown above and below the branches, respectively. “*” indicates the node not found in the strict consensus tree. Tamura’s [19] classification is shown on the right.
(TIF)

S1 Table Taxa, voucher identification, locality, and GenBank accession numbers for molecular analyses of the genus *Coptis*. “–” indicates data not available. “*” means newly generated sequences in this study.
(DOC)

S2 Table Morphological characters and states on which the taxonomy of *Coptis* has been mostly based. (DOC)

S3 Table Pairwise divergence of *trnL-F* sequences from *Coptis*. (DOC)

S4 Table Pairwise divergence of *trnH-psbA* sequences from *Coptis*. (DOC)

S5 Table Pairwise divergence of ITS sequences from *Coptis*. (DOC)
