# Supplementary figures and images for: The First Comprehensive Phylogeny of Coptis (Ranunculaceae) and Its Implications for Character Evolution and Classification
Source: PLoS One. 2016 Apr 4;11(4):e0153127. doi: 10.1371/journal.pone.0153127 (PMC4820238; doi:10.1371/journal.pone.0153127)

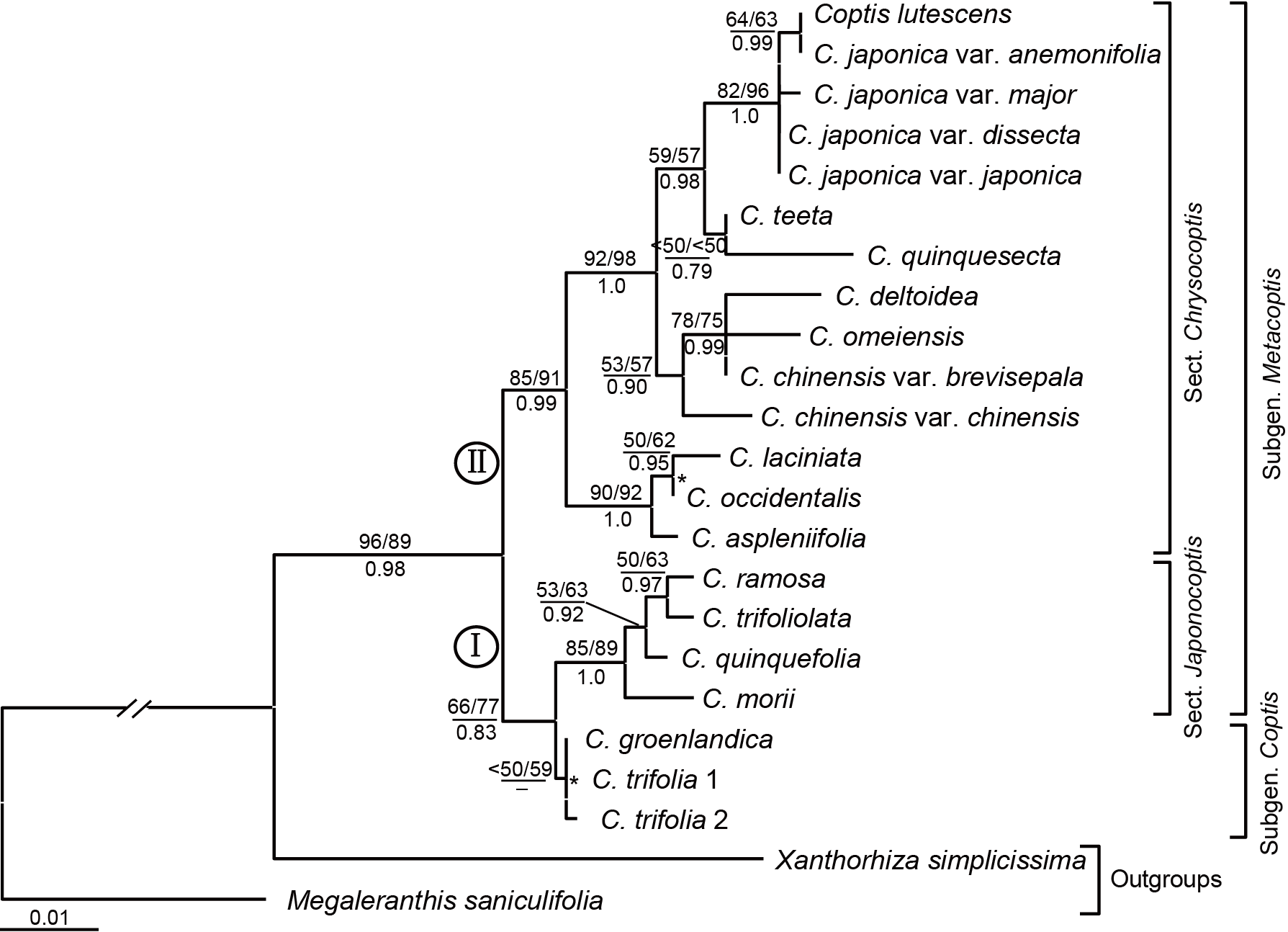

Supplement: S1 Fig — The results of MP and ML bootstrap analyses are shown above the branches, whereas the values below the branches result from Bayesian analysis. “*” indicates the nodes not found in the strict consensus tree. Tamura’s [19] classification is shown on the right. (TIF) [file pone.0153127.s001.tif]

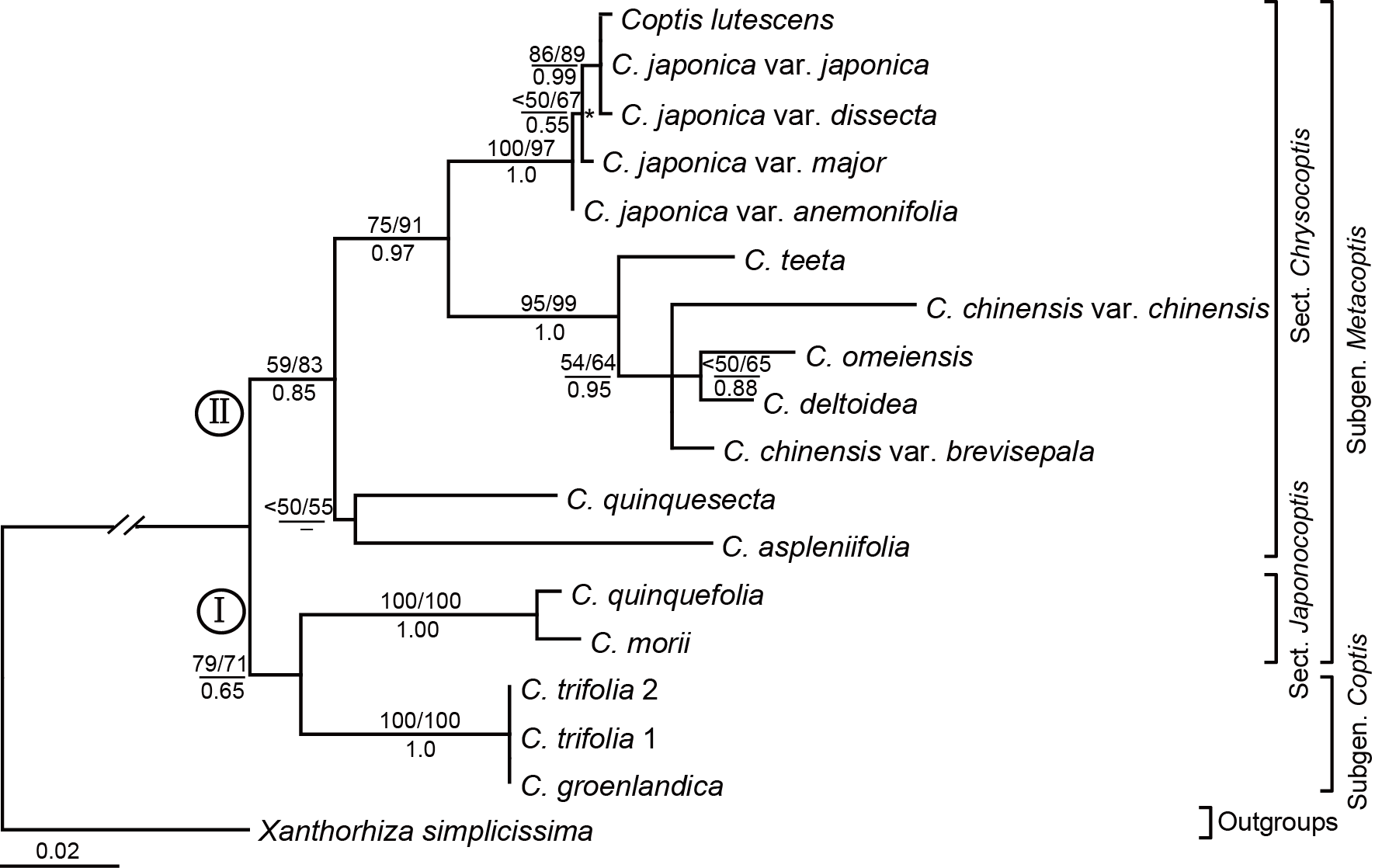

Supplement: S2 Fig — The results of MP and ML bootstrap analyses are shown above the branches, whereas the values below the branches result from Bayesian analysis. “*” indicates the node not found in the strict consensus tree. Tamura’s [19] classification is shown on the right. (TIF) [file pone.0153127.s002.tif]

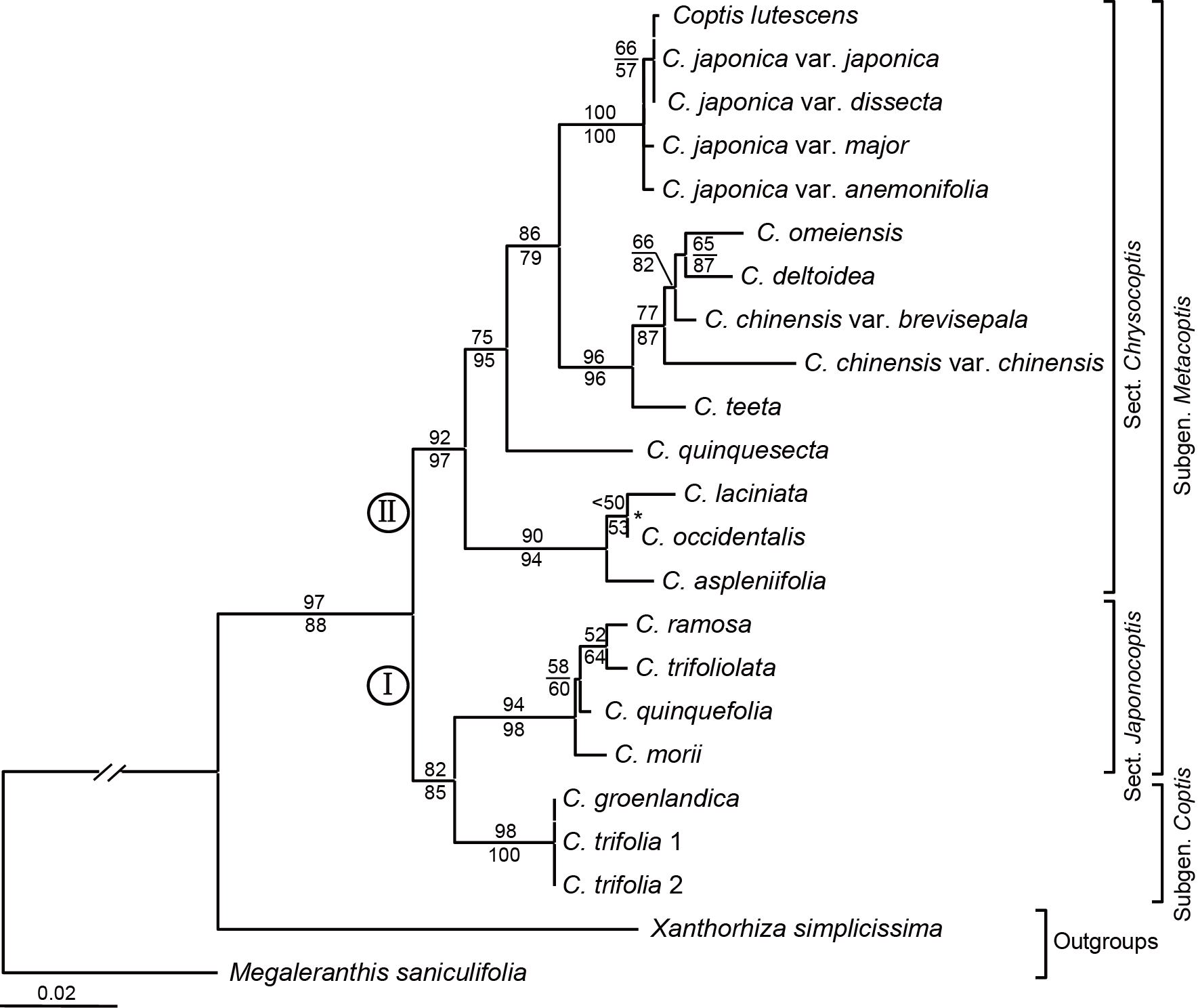

Supplement: S3 Fig — The results of MP and ML bootstrap analyses are shown above and below the branches, respectively. “*” indicates the node not found in the strict consensus tree. Tamura’s [19] classification is shown on the right. (TIF) [file pone.0153127.s003.tif]
