# Supplementary material for: The First Comprehensive Phylogeny of Coptis (Ranunculaceae) and Its Implications for Character Evolution and Classification
Source: PLoS One. 2016 Apr 4;11(4):e0153127. doi: 10.1371/journal.pone.0153127 (PMC4820238; doi:10.1371/journal.pone.0153127)
Supplement: S1 Table — “–” indicates data not available. “*” means newly generated sequences in this study. (DOC) [file pone.0153127.s004.doc]

**S1 Table. Species names and GenBank accession numbers of DNA sequences used in this study.** “–” indicates data not available. “*” means newly generated sequences in this study.

|  |  |  | Genbank accession numbers | | |  |
| --- | --- | --- | --- | --- | --- | --- |
| Species | Voucher (Herbarium) | Locality | *trnL-F* | *trnH-psbA* | ITS | References |
| **Ingroups** |  |  |  |  |  |  |
| *Coptis aspleniifolia* Salisb. | Jim Bjar 45 (PE) | Vancouver Island, USA | AB163738 | AB163747 | *KR697579 | [This study, 1] |
| *Coptis chinensis* Franch. | – | – | AB163736 | JN862867 | JN862853 | [1,2] |
| *Coptis chinensis* var. *brevisepala* W.T. Wang & P.G. Xiao | – | – | – | HQ829530 | JN862855 | [2,3] |
| *Coptis deltoidea* C.Y. Cheng & P.G. Xiao | – | – | AB163735 | JN862873 | JN862856 | [1,2] |
| *Coptis groenlandica* (O.F. Müll.) Fernald | EW Wood & DE Bonfford 4720 (PE) | Caledonia, USA | *KR697590 | – | *KR697582 | [This study] |
| *Coptis japonica* var. *anemonifolia* (Siebold & Zucc.) H. Ohba | Takenchi T 525 (PE) | Japan | *KR697588 | AB163752 | AB695574 | [1,4] |
| *Coptis japonica* var. *dissecta* (Yatabe) Nakai ex Satake | S Tsugam & T Takahashii 23196 (PE) | Yamanashi, Japan | *KR697589 | *KR697586 | *KR697578 | [This study] |
| *Coptis japonica* var. *japonica* (Thunb.) Makino | – | – | – | AB163751 | AB695605 | [1,4] |
| *Coptis japonica* var. *major* (Miq.) Satake | – | – | AB163742 | AB163753 | AB695583 | [1,4] |
| *Coptis laciniata* A. Gray | – | – | AB163739 | AB163748 | – | [1] |
| *Coptis lutescens* Tamura | – | – | AB159517 | AB159519 | AB695607 | [1,4] |
| *Coptis morii* Hayata | Chen et al. 20110736 (PE) | Mt. Poluo, Yilan County, Taiwan | *KR697587 | *KR697584 | EF206702 | [This study, 5] |
| *Coptis occidentalis* (Nutt.) Torr. & A. Gray | – | – | AB163740 | AB163749 | – | [1] |
| *Coptis omeiensis* (Chen) C.Y. Cheng | – | – | AB163737 | JN862874 | HQ829630 | [1,2,3] |
| *Coptis quinquefolia* Miq. | G Mwrota & T Takahashi 29031 (PE) | Japan | AB159522 | *KR697585 | *KR697577 | [This study, 1] |
| *Coptis quinquesecta* W.T. Wang | Anonymous 2471 (PE) | Jinping County, Yunnan | AB159546 | AB159545 | *KR697580 | [This study, 1] |
| *Coptis ramosa* (Makino) Tamura | – | – | AB159535 | AB159534 | – | [1] |
| *Coptis teeta* Wall. | – | – | AB163734 | JN862876 | JN862858 | [1,2] |
| *Coptis trifolia* (L.) Salisb.^1^ | – | – | AB159540 | AB159539 | AB695610 | [1,4] |
| *Coptis trifolia* (L.) Salisb.^2^ | Ying et al. 1299 (PE) | Caledonia, USA | AB159542 | AB159541 | *KR697581 | [This study, 1] |
| *Coptis trifoliolata*(Makino) Makino | – | – | AB159531 | AB159530 | – | [1] |
| **Outgroups** |  |  |  |  |  |  |
| *Xanthorhiza simplicissima* Marshall | SR Hill 22188 (PE) | Tennessee, USA | EF437111 | AB163750 | *KR697583 | [1,6] |
| *Megaleranthis saniculifolia* Ohwi | – | – | HQ440188 | NC012615 | – | [7,8] |

**References**

1. Terunuma Y, Kondo K, Shiba M, Amagaya S, Aburada M, Setoguchi H. Phylogeny of *Coptis* (Ranunculaceae). Unpublished.
2. Liu XG, Yuan QJ, Song LK, Huang LQ. Study on DNA barcoding in the medicinal plants of *Coptis* Salisb. Unpublished.
3. Liu XG, Yuan QJ, Song LK, Huang LQ. Study on molecular systematics in the medicinal plants of Coptis. Unpublished.
4. Shinozaki J, Shirota O, Masuda K, Satake M, Sekita S, Takano A. Phylogenetic analysis of *Coptis japonica* inferred from plastid *matK* and nuclear ribosomal ITS sequences. Unpublished.
5. Lin TC, Wu CT, Hsieh CC. Identification of *Coptis* species based on ITS sequence of nuclear ribosomal DNA in Taiwan. Unpublished.
6. Wang W, Chen ZD (2007) Generic level phylogeny of Thalictroideae (Ranunculaceae) – implications for the taxonomic status of *Paropyrum* and petal evolution. Taxon 56: 811–821
7. Wang W, Hu H, Xiang XG, Yu SX, Chen ZD (2010) Phylogenetic placements of *Calathodes* and *Megaleranthis* (Ranunculaceae): Evidence from molecular and morphological data. Taxon 59: 1712–1720.
8. Kim YK, Park CW, Kim KJ (2009) Complete Chloroplast DNA Sequence from a Korean Endemic Genus, *Megaleranthis saniculifolia*, and Its Evolutionary Implications. Mol Cells 27: 365–381.
