# Supplementary material for: The First Comprehensive Phylogeny of Coptis (Ranunculaceae) and Its Implications for Character Evolution and Classification
Source: PLoS One. 2016 Apr 4;11(4):e0153127. doi: 10.1371/journal.pone.0153127 (PMC4820238; doi:10.1371/journal.pone.0153127)
Supplement: S2 Table — (DOC) [file pone.0153127.s005.doc]

**S2 Table. Morphological characters and states on which the taxonomy of *Coptis* has been mostly based.**

|  | Rhizome | Basal leaf type | No. leaflets | Central leaflet base (length in mm) | Lateral leaflet base (length in cm) | Length/width of central leaflet | Size of lateral and central leaflets | Scapes branched | No. flowers | Bract shape | |
| --- | --- | --- | --- | --- | --- | --- | --- | --- | --- | --- | --- |
|
| **Subgen. *Metacoptis*** | |  |  |  |  |  |  |  |  |  | |
| **Sect. *Chrysocoptis*** |  |  |  |  |  |  |  |  |  |  | |
| *C. chinensis* | yellow | ternate | 3 | petiolate | subsessile (< 0.5) | >1.5 | unequal | yes | 3–8 | lanceolate | |
| *C. deltoidea* | yellow | ternate | 3 | petiolate | subsessile (< 0.5) | >1.5 | unequal | yes | 4–8 | linear-lanceolate | |
| *C. japonica* | – | 2-, 3-ternate | ≥ 9 | petiolate | petiolate | >1.5 | unequal | yes | 3–4 | linear-lanceolate | |
| *C. lutescens* | – | 2- to 4-ternate | ≥ 9 | petiolate | petiolate | >1.5 | unequal | yes | 3–4 | linear-lanceolate | |
| *C. omeiensis* | yellow | ternate | 3 | petiolate | subsessile (< 0.5) | >1.5 | unequal | yes | 4 | lanceolate | |
| *C. quinquesecta* | yellow | pedate | 5 | petiolate | subsessile (< 0.5) | >1.5 | unequal | yes | 6 | lanceolate | |
| *C. teeta* | yellow | ternate | 3 | petiolate | subsessile (< 0.5) | >1.5 | unequal | yes | 3–4 (-5) | elliptic | |
| *C. laciniata* | pale brown | 1, 2-ternate | 3, 9 | petiolate | petiolate | 1 | equal | yes | 2–4 | – | |
| *C. occidentalis* | pale brown | ternate | 3 | petiolate | petiolate | 1 | equal | yes | 2–3 (-5) | | – |
| *C. aspleniifolia* | pale brown | 2-, 3-pinnate or 2-ternate | ≥ 9 | petiolate | petiolate | 1 | equal | yes | 2–3 | – | |
| **Sect.** ***Japonocoptis*** |  |  |  |  |  |  |  |  |  |  | |
| *C. morii* | yellow | pedate | 5 | subsessile (< 0.5) | sessile | 1 | equal | yes | ca. 4 | lanceolate | |
| *C. quinquefolia* | yellow | pedate | 5 | sessile | sessile | 1 | equal | no | 1 | elliptic | |
| *C. ramosa* | – | pedate | 5 | sessile | sessile | 1 | equal | no | 1–3 | elliptic | |
| *C. trifoliolata* | – | ternate | 3 | sessile | sessile | 1 | equal | no | 1 | elliptic | |
| **Subgen. *Coptis*** |  |  |  |  |  |  |  |  |  |  | |
| *C. trifolia* | bright yellow to orange | ternate | 3 | subsessile (< 0.5) | subsessile (< 0.5) | 1 | equal | no | 1 | elliptic | |
| **Sister to *Coptis*** |  |  |  |  |  |  |  |  |  |  | |
| *Xanthorhiza* | yellow | 1-, 2-pinnate | 5 | petiolate | subsessile (< 0.5) | 1 | equal | yes | many | scalelike | |

(*Cont.*)

|  | Bract margin | Sepal color | Sepal shape | No. petals | Petal length (mm) | Petal color | Petal shape | Pattern of petal blade attachment | Beak length (mm) |
| --- | --- | --- | --- | --- | --- | --- | --- | --- | --- |
|
| **Subgen. *Metacoptis*** | |  |  |  |  |  |  |  |  |
| **Sect. *Chrysocoptis*** |  |  |  |  |  |  |  |  |  |
| *C. chinensis* | palmately divided | greenish yellow | lanceolate | 10 | 5–6.5 | greenish yellow | linear-lanceolate | epeltate | < 1 |
| *C. deltoidea* | 3-parted or pinnately divided | greenish yellow | lanceolate | 10 | 3–6.5 | greenish yellow | linear-lanceolate | epeltate | < 1 |
| *C. japonica* | 3-lobed | white | narrowly lanceolate | 8-10 | ~4 | white | spatulate | epeltate | < 1 |
| *C. lutescens* | 3-lobed | greenish yellow, sometimes white | narrowly lanceolate to linear | 8-11 | ~4 | yellow, sometimes white or rarely purplish | spatulate | epeltate | < 1 |
| *C. omeiensis* | finely serrate | greenish yellow | narrowly lanceolate | 9-12 | 4–5 | greenish yellow | linear-lanceolate | epeltate | < 1 |
| *C. quinquesecta* | 3-lobed or acutely serrate | greenish yellow | lanceolate | 8-10 | ? | greenish yellow | linear-lanceolate | epeltate | < 1 |
| *C. teeta* | 3-parted or pinnately divided | greenish yellow | lanceolate | 10 | 4.5–5.9 | greenish yellow | spatulate | epeltate | < 1 |
| *C. laciniata* | – | greenish white | lanceolate to linear | 5-7 | – | greenish white | linear-lanceolate | epeltate | < 1 |
| *C. occidentalis* | – | greenish white | lanceolate to linear | 5-7 | – | greenish white | linear-lanceolate | epeltate | < 1 |
| *C. aspleniifolia* | – | greenish white | lanceolate to linear | 5-7 | – | greenish white | linear-lanceolate | epeltate | < 1 |
| **Sect. *Japonocoptis*** |  |  |  |  |  |  |  |  |  |
| *C. morii* | acute serrate | white | elliptic | 5 | 1.6–3 | yellow | cup-shaped | peltate | < 1 |
| *C. quinquefolia* | entire | white | elliptic |  | 2–4 | yellow | cup-shaped | peltate | < 1 |
| *C. ramosa* | entire | white | elliptic | 5 | ~3 | yellow | cup-shaped | peltate | < 1 |
| *C. trifoliolata* | entire | white | elliptic | 5 (or 6) | 3–4 | yellow | cup-shaped | peltate | < 1 |
| **Subgen. *Coptis*** |  |  |  |  |  |  |  |  |  |
| *C. trifolia* | entire | white | elliptic | 5 | 2–3 | yellow | cup-shaped | peltate | 2–4 |
| **Sister to *Coptis*** |  |  |  |  |  |  |  |  |  |
| *Xanthorhiza* | entire | brownish purple | elliptic | 5 |  | brownish purple | clavate | peltate | 0.4–0.8 |
