# Supplementary material for: The First Comprehensive Phylogeny of Coptis (Ranunculaceae) and Its Implications for Character Evolution and Classification
Source: PLoS One. 2016 Apr 4;11(4):e0153127. doi: 10.1371/journal.pone.0153127 (PMC4820238; doi:10.1371/journal.pone.0153127)
Supplement: S4 Table — (DOC) [file pone.0153127.s007.doc]

**S4 Table. Pairwise divergence of *trnH-psbA* sequences from *Coptis***.

| Taxon | 1 | 2 | 3 | 4 | 5 | 6 | 7 | 8 | 9 | 10 | 11 | 12 | 13 | 14 | 15 | 16 | 17 | 18 | 19 | 20 |
| --- | --- | --- | --- | --- | --- | --- | --- | --- | --- | --- | --- | --- | --- | --- | --- | --- | --- | --- | --- | --- |
| 1 *C. aspleniifolia* | – |  |  |  |  |  |  |  |  |  |  |  |  |  |  |  |  |  |  |  |
| 2 *C. chinensis* | 0.022 | – |  |  |  |  |  |  |  |  |  |  |  |  |  |  |  |  |  |  |
| 3 *C. chinensis* var. *brevisepala* | 0.007 | 0.015 | – |  |  |  |  |  |  |  |  |  |  |  |  |  |  |  |  |  |
| 4 *C. deltoidea* | 0.015 | 0.022 | 0.007 | – |  |  |  |  |  |  |  |  |  |  |  |  |  |  |  |  |
| 5 *C. japonica* var. *anemonifolia* | 0.030 | 0.037 | 0.022 | 0.030 | – |  |  |  |  |  |  |  |  |  |  |  |  |  |  |  |
| 6 *C. japonica* var. *dissecta* | 0.022 | 0.030 | 0.015 | 0.022 | 0.007 | – |  |  |  |  |  |  |  |  |  |  |  |  |  |  |
| 7 *C. japonica* var. *japonica* | 0.022 | 0.030 | 0.015 | 0.022 | 0.007 | 0.000 | – |  |  |  |  |  |  |  |  |  |  |  |  |  |
| 8 *C. japonica* var. *major* | 0.030 | 0.037 | 0.022 | 0.030 | 0.015 | 0.007 | 0.007 | – |  |  |  |  |  |  |  |  |  |  |  |  |
| 9 *C. laciniata* | 0.000 | 0.022 | 0.007 | 0.015 | 0.030 | 0.022 | 0.022 | 0.030 | – |  |  |  |  |  |  |  |  |  |  |  |
| 10 *C. lutescens* | 0.030 | 0.037 | 0.022 | 0.030 | 0.000 | 0.007 | 0.007 | 0.015 | 0.030 | – |  |  |  |  |  |  |  |  |  |  |
| 11 *C. occidentalis* | 0.000 | 0.022 | 0.007 | 0.015 | 0.030 | 0.022 | 0.022 | 0.030 | 0.000 | 0.030 | – |  |  |  |  |  |  |  |  |  |
| 12 *C. omeiensis* | 0.015 | 0.022 | 0.007 | 0.015 | 0.030 | 0.022 | 0.022 | 0.030 | 0.015 | 0.030 | 0.015 | – |  |  |  |  |  |  |  |  |
| 13 *C. quinquefolia* | 0.015 | 0.030 | 0.015 | 0.022 | 0.037 | 0.030 | 0.030 | 0.037 | 0.015 | 0.037 | 0.015 | 0.022 | – |  |  |  |  |  |  |  |
| 14 *C. morii* | 0.015 | 0.030 | 0.015 | 0.022 | 0.037 | 0.030 | 0.030 | 0.037 | 0.015 | 0.037 | 0.015 | 0.022 | 0.015 | – |  |  |  |  |  |  |
| 15 *C. quinquesecta* | 0.022 | 0.030 | 0.015 | 0.022 | 0.022 | 0.015 | 0.015 | 0.022 | 0.022 | 0.022 | 0.022 | 0.022 | 0.030 | 0.030 | – |  |  |  |  |  |
| 16 *C. ramosa* | 0.022 | 0.037 | 0.022 | 0.030 | 0.045 | 0.037 | 0.037 | 0.045 | 0.022 | 0.045 | 0.022 | 0.030 | 0.007 | 0.007 | 0.037 | – |  |  |  |  |
| 17 *C. teeta* | 0.015 | 0.022 | 0.007 | 0.015 | 0.015 | 0.007 | 0.007 | 0.015 | 0.015 | 0.015 | 0.015 | 0.015 | 0.022 | 0.022 | 0.007 | 0.030 | – |  |  |  |
| 18 *C. trifolia* (USA) | 0.007 | 0.030 | 0.015 | 0.022 | 0.037 | 0.030 | 0.030 | 0.037 | 0.007 | 0.037 | 0.007 | 0.022 | 0.007 | 0.022 | 0.030 | 0.015 | 0.022 | – |  |  |
| 19 *C. trifolia* (Japan) | 0.007 | 0.030 | 0.015 | 0.022 | 0.037 | 0.030 | 0.030 | 0.037 | 0.007 | 0.037 | 0.007 | 0.022 | 0.007 | 0.022 | 0.030 | 0.015 | 0.022 | 0.000 | – |  |
| 20 *C. trifoliolata* | 0.015 | 0.030 | 0.015 | 0.022 | 0.037 | 0.030 | 0.030 | 0.037 | 0.015 | 0.037 | 0.015 | 0.022 | 0.000 | 0.015 | 0.030 | 0.007 | 0.022 | 0.007 | 0.007 | – |
