# Supplementary material for: The First Comprehensive Phylogeny of Coptis (Ranunculaceae) and Its Implications for Character Evolution and Classification
Source: PLoS One. 2016 Apr 4;11(4):e0153127. doi: 10.1371/journal.pone.0153127 (PMC4820238; doi:10.1371/journal.pone.0153127)
Supplement: S5 Table — (DOC) [file pone.0153127.s008.doc]

**S5 Table. Pairwise divergence of ITS sequences from *Coptis***.

| Taxon | 1 | 2 | 3 | 4 | 5 | 6 | 7 | 8 | 9 | 10 | 11 | 12 | 13 | 14 | 15 | 16 | 17 |
| --- | --- | --- | --- | --- | --- | --- | --- | --- | --- | --- | --- | --- | --- | --- | --- | --- | --- |
| 1 *C. aspleniifolia* | – |  |  |  |  |  |  |  |  |  |  |  |  |  |  |  |  |
| 2 *C. chinensis* | 0.120 | – |  |  |  |  |  |  |  |  |  |  |  |  |  |  |  |
| 3 *C. chinensis* var. *brevisepala* | 0.106 | 0.063 | – |  |  |  |  |  |  |  |  |  |  |  |  |  |  |
| 4 *C. deltoidea* | 0.101 | 0.071 | 0.028 | – |  |  |  |  |  |  |  |  |  |  |  |  |  |
| 5 *C. groenlandica* | 0.135 | 0.124 | 0.120 | 0.110 | – |  |  |  |  |  |  |  |  |  |  |  |  |
| 6 *C. japonica* var. *anemonifolia* | 0.093 | 0.102 | 0.070 | 0.079 | 0.097 | – |  |  |  |  |  |  |  |  |  |  |  |
| 7 *C. japonica* var. *dissecta* | 0.098 | 0.093 | 0.079 | 0.088 | 0.115 | 0.016 | – |  |  |  |  |  |  |  |  |  |  |
| 8 *C. japonica* var. *japonica* | 0.102 | 0.088 | 0.075 | 0.084 | 0.111 | 0.012 | 0.004 | – |  |  |  |  |  |  |  |  |  |
| 9 *C. japonica* var. *major* | 0.098 | 0.098 | 0.066 | 0.075 | 0.102 | 0.004 | 0.012 | 0.008 | – |  |  |  |  |  |  |  |  |
| 10 *C. lutescens* | 0.102 | 0.088 | 0.075 | 0.084 | 0.111 | 0.012 | 0.004 | 0.000 | 0.008 | – |  |  |  |  |  |  |  |
| 11 *C. omeiensis* | 0.093 | 0.067 | 0.028 | 0.032 | 0.134 | 0.084 | 0.093 | 0.089 | 0.079 | 0.089 | – |  |  |  |  |  |  |
| 12 *C. quinquefolia* | 0.116 | 0.146 | 0.098 | 0.107 | 0.093 | 0.098 | 0.117 | 0.113 | 0.103 | 0.113 | 0.122 | – |  |  |  |  |  |
| 13 *C. morii* | 0.112 | 0.151 | 0.102 | 0.111 | 0.098 | 0.103 | 0.113 | 0.117 | 0.108 | 0.117 | 0.126 | 0.020 | – |  |  |  |  |
| 14 *C. quinquesecta* | 0.084 | 0.117 | 0.103 | 0.084 | 0.098 | 0.085 | 0.103 | 0.099 | 0.089 | 0.099 | 0.089 | 0.118 | 0.123 | – |  |  |  |
| 15 *C. teeta* | 0.084 | 0.058 | 0.028 | 0.041 | 0.135 | 0.075 | 0.084 | 0.080 | 0.071 | 0.080 | 0.016 | 0.112 | 0.117 | 0.080 | – |  |  |
| 16 *C. trifolia* (USA) | 0.135 | 0.124 | 0.120 | 0.110 | 0.000 | 0.097 | 0.115 | 0.111 | 0.102 | 0.111 | 0.134 | 0.093 | 0.098 | 0.098 | 0.135 | – |  |
| 17 *C. trifolia* (Japan) | 0.135 | 0.124 | 0.120 | 0.110 | 0.000 | 0.097 | 0.115 | 0.111 | 0.102 | 0.111 | 0.134 | 0.093 | 0.098 | 0.098 | 0.135 | 0.000 | – |
